# Neuroprotection by canagliflozin in a Huntington’s disease model: role of HIF-1α and PI3K/AKT signaling

**DOI:** 10.1007/s00210-025-04891-5

**Published:** 2025-12-26

**Authors:** Ali M. Elgindy, El-Sayed E. El-Awady, Norhan M. El-Sayed, Naglaa F. El-Orabi, Ahmed M. Atwa

**Affiliations:** 1 Department of Pharmacology and Toxicology, Faculty of Pharmacy, Egyptian Russian University, Cairo, 11829 Egypt; 2 Department of Pharmacology and Toxicology, Faculty of Pharmacy, Suez Canal University, Ismailia, 41522 Egypt; 3 College of Pharmacy, Al-Ayen Iraqi University, AUIQ, An Nasiriyah, Iraq

**Keywords:** 3-Nitropropionic acid, Neurodegeneration, Canagliflozin, PI3K/AKT signaling, HIF-1α

## Abstract

Huntington’s disease (HD) is a progressive neurodegenerative disorder characterized by motor, cognitive, and metabolic dysfunction, largely driven by mitochondrial impairment and defective energy metabolism. Altered signaling through hypoxia-inducible factor-1α (HIF-1α) and PI3K/AKT cascades contributes to neuronal vulnerability. Canagliflozin (Cana), a sodium–glucose cotransporter-2 inhibitor, has shown cognitive benefits in experimental studies. Here, we evaluated whether Cana mitigates 3-nitropropionic acid (3NP, 10 mg/kg, i.p., 14 days)-induced HD-like neurotoxicity in rats. Animals received Cana (5 or 10 mg/kg, p.o.) daily for 14 days, followed by behavioral assessments (open-field, Morris water maze, novel object recognition), histopathology, immunohistochemistry, and biochemical assays. Cana treatment significantly improved locomotor and memory performance, reduced striatal histopathological alterations, and attenuated GFAP immunoreactivity. Mechanistically, Cana upregulated HIF-1α and downstream GLUT1/GLUT3/HKII, restored PI3K/AKT/CREB/BDNF signaling, and enhanced SIRT1/PGC-1α/Nrf2 antioxidant responses, while suppressing inflammatory mediators and caspase-3 activation. These findings highlight Cana as a promising disease-modifying strategy for HD by targeting both energy metabolism and pro-survival pathways.

**Supplementary Information:**

The online version contains supplementary material available at 10.1007/s00210-025-04891-5.

## Introduction

Huntington's disease (HD) is an autosomal dominant neurodegenerative condition marked by a gradual deterioration of motor, cognitive, and mental abilities, with symptoms generally manifesting between the ages of 30 and 40 (Tramutola et al. 2023). The precise etiology of neuronal degeneration in Huntington's disease remains inadequately elucidated. Oxidative stress, mitochondrial dysfunction, and disturbances in energy metabolism are acknowledged as significant factors in its etiology (Burtscher et al. 2023).

Mitochondrial dysfunction and energy metabolism manifest in several ways including glucose hypometabolism in the brain (Squitieri and Ciarmiello 2010), impairments in oxidative phosphorylation (Milakovic and Johnson 2005), mitochondrial dysfunction (Li et al. 2010), oxidative stress (Olufunmilayo et al. 2023), and dysfunction of peroxisome proliferator-activated receptor gamma co-activator-1-alpha (PGC-1α), the principal regulator of mitochondrial biogenesis (Tabrizi et al. 2020).

The brain depends on glucose for metabolic processes, facilitating cellular functioning and neurotransmitter production (Mergenthaler et al. 2013). Glucose infiltrates neuronal cells through glucose transporter (GLUT) proteins, predominantly GLUT1 and GLUT3, which are the most prevalent in the brain (Daida et al. 2024). Impaired glucose absorption, especially in neurons, contributes to energy metabolic deficiencies in HD patients (Adanyeguh et al. 2015). Decreased expression of GLUT1 and GLUT3 has been observed in neurodegenerative diseases like HD (Morea et al. 2017), Epilepsy (Patanè 2021), and Alzheimer’s disease (AD) (Kyrtata et al. 2021).

Furthermore, hypoxia-inducible factor 1-alpha (HIF-1α), a pivotal regulatory gene for GLUT1 and GLUT3 production (Zhang et al. 2011), has a vital function as a neuroprotective agent (Ashok et al. 2017). The overexpression of HIF-1α may potentially diminish the risk of neurodegenerative disorders, including AD (Ke and Costa 2006), Parkinson disease (PD) (Lee et al. 2009), and HD (Yang et al. 2005).

The induction of HIF-1α expression increased the amounts of glucose transporters GLUT1 and GLUT3, together with glycolytic enzymes, hence augmenting glucose absorption in hypoxic neurons (Wu et al. 2021). This enhanced energy provision facilitates neurogenesis and diminishes neuronal mortality (Wu et al. 2021). Furthermore, HIF-1α directly engages with hexokinases, specifically hexokinase II (HKII), which facilitates the initial phase of glycolysis, hence enhancing mitochondrial control (Pastorino and Hoek 2008).

The phosphoinositide 3-kinase (PI3K) and protein kinase B (AKT) pathway is essential in the management of several neurodegenerative illnesses, principally by enhancing cell proliferation and modulating several downstream molecules associated with cell survival and neuroprotection (Vaillant et al. 1999). The PI3K/AKT pathway activates cAMP-responsive element-binding protein (CREB), hence enhancing the production of brain-derived neurotrophic factor (BDNF). Brain-derived neurotrophic factor (BDNF) facilitates neurogenesis, inhibits apoptosis, and augments synaptic plasticity (Wang et al. 2016; Xia et al. 2010). The PI3K/AKT pathway also facilitates glucose absorption and energy metabolism by modulating the expression of the GLUT3 transporter, therefore improving local glucose metabolism (Agostini et al. 2016; McNay et al. 2010).

A significant downstream effector of the PI3K/AKT pathway is sirtuin 1 (SIRT1) (Yang et al. 2021). Studies has shown that SIRT1 activation has substantial neuroprotective benefits in neurodegenerative disorders (Kim et al. 2007), in addition to its role in cell cycle regulation, DNA repair, neurogenesis, and metabolism (Lee et al. 2018). SIRT1 interacts with and deacetylates PGC-1α, thereby augmenting its activity, facilitating mitochondrial biogenesis, and preserving mitochondrial function (Lagouge et al. 2006). Additionally, SIRT1 demonstrates a neuroprotective impact by enhancing the transcriptional activity of nuclear factor erythroid 2-related factor 2 (Nrf2), reducing oxidative stress (Ren et al. 2019), and stimulation of BDNF expression (Tian et al. 2020).

3-Nitropropionic acid (3NP), a neurotoxin with striatal selectivity, emulates Huntington's disease pathophysiology by blocking succinate dehydrogenase, hence inducing oxidative stress, (Ranju et al. 2015), mitochondrial failure, neuroinflammation, and excitotoxicity (Mehan et al. 2017). These disruptions hinder energy metabolism, diminish ATP levels, and result in neural malfunction, cellular mortality, and behavioral impairments in rats (Orozco-Ibarra et al. 2018).

SGLT2 inhibitors, mostly utilized for type 2 diabetes, lower blood glucose levels by blocking SGLT2 receptors (Hsia et al. 2017). Importantly, they cross the blood–brain barrier and have shown neuroprotective, partly by mitigating oxidative stress and inflammation and by enhancing HIF-1α expression (Hierro-Bujalance et al. 2020). Canagliflozin (Cana), an SGLT2 inhibitor, demonstrates potential in averting memory deterioration by activating the PI3K/AKT pathway and upregulating BDNF, which may alleviate cognitive decline (Huang et al. 2024). Given the elevated frequency of diabetes among HD patients, Canagliflozin may provide dual advantages for diabetes management and neuroprotection in HD (Chaves et al. 2019).

Given the emerging neuroprotective effects of SGLT2 inhibitors and the metabolic impairments observed in HD, this study investigates the therapeutic potential of Canagliflozin in a 3NP-induced rat model of HD. We hypothesize that Canagliflozin attenuates neurotoxicity by modulating the HIF-1α/GLUT and PI3K/AKT/CREB/BDNF pathways.

## Material and methods

### Animals

A total of 60 male Wistar albino rats (weight range: 170–200 g) were procured from The Nile for Pharmaceuticals & Chemical Industries and housed at the animal facility of the Faculty of Pharmacy at Suez Canal University for a one-week acclimatization period. The housing environment maintained at a consistent temperature of 23 ± 2 °C with a 12-h light/dark cycle and had unrestricted access to food and water. Animal care as well as all experimental procedures were undertaken in compliance with the specified guidelines of the Animal Care and Use Committee of the Faculty of Pharmacy, Suez Canal University (Serial number of the protocol: 202,302 PhDA_1_).

### Experimental design

Canagliflozin and 3-Nitropropionic acid obtained from (Sigma-Aldrich, USA), were freshly prepared daily by dissolving in normal saline with pH neutralized at 7.4 using NaOH. Rats were divided randomly into 5 groups (12 rats per group). Group 1: injected with normal saline (i.p) daily for 14 days. Group 2: injected with Canagliflozin (10 mg/kg, p.o.) daily for 14 days (Arafa et al. 2016). Group (3–5): injected with 3NP (10 mg/kg, i.p) daily for 14 days (Danduga et al. 2018). Group 3 was considered the induction group. Group (4–5): injected with Canagliflozin (5 or 10 mg/kg, p.o.) 1 h after 3NP, respectively.

**Scheme 1:**
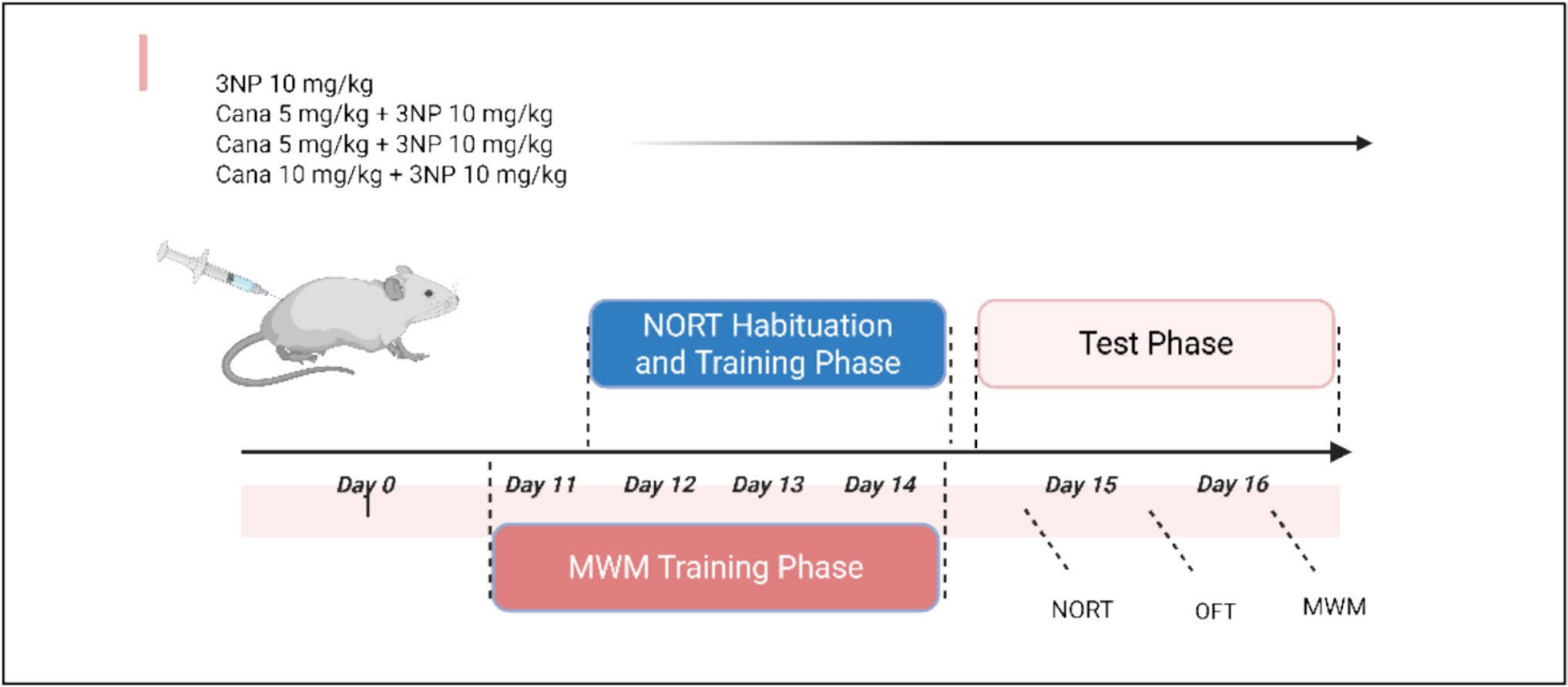
Sequence for experimental design, behavioral assessment. 3NP, 3-nitropropionic acid; Cana, Canagliflozin

Drugs were given for 14 days and rats were subjected to the behavioral tests on the 15th and 16th days (Scheme 1). Animals were then euthanized using I.P. injection of 100 mg/kg ketamine and 10 mg/kg xylazine, then sacrificed by cervical dislocation. Brains were dissected with striata being separated. Brain samples were processed for the histopathological and immunohistochemical examinations in addition to the estimation of biochemical parameters in the striatal region.

### Behavioral assessment

#### Open field test (OPT)

The impulsive locomotor activity of the animals was evaluated using a square wooden box measuring 80 × 80 × 40 cm, featuring red walls and a black polished floor divided into 16 equal squares by white lines. The experiment took place in a soundproof room, and the rats' movements were recorded via an overhead camera. Each rat was placed in the center of the box and allowed to explore freely for a duration of 5 min.

Several parameters were assessed during this period, the ambulation frequency, which defines the square number each rat has crossed, and the number of rearing events, which defines the number of stretches on the rat’s hind limbs. Those often reflect exploratory behavior and locomotor function, offering further insight into the animals' activity levels and impulsive behavior. This setup provided a comprehensive analysis of the rats' spontaneous motor activity and exploratory patterns (Ramachandran and Thangarajan 2018).

#### Morris water maze test (MWM)

The Morris water maze test was employed to assess spatial learning and memory retention in rats. The experiment required training the animals to locate a submerged platform within a circular pool measuring 150 cm in diameter and 60 cm in height. The pool, featuring non-reflective inner walls, was partitioned into four quadrants and filled with water to a depth of 35 cm, kept at a temperature of 25 ± 2 °C. During the acquisition phase, a moveable platform with a diameter of 9 cm was positioned 1 cm beneath the water surface in a designated quadrant.

The training comprised three daily sessions, each lasting 120 s, conducted over four days. In these sessions, rats were permitted to locate the platform from different beginning positions. If a rat could not find the platform, it was directed to it and let to rest for 30 s. Subsequent to the training phase, the test was conducted by extracting the platform from the water. The rat was subsequently positioned in the pool, orientated towards the wall in a quadrant opposite to the prior location of the platform. The water was mixed with non-toxic, soluble black dye to diminish visibility. The animal was permitted one minute for exploration, during which its swimming duration in the target quadrant was recorded via an above camera (Suganya and Sumathi 2017).

#### Novel object recognition test (NORT)

The novel object recognition test was conducted to assess cognitive function, particularly recognition memory. The experiment took place in a black open field box measuring 50 × 25 × 50 cm. During the habituation phase, the rats were allowed to explore the empty box for 10 min per day over two consecutive days, familiarizing them with the environment.

During the training sessions, each rat was introduced into a box containing two identical objects placed in opposite corners, spaced about 30 cm distant. On the test day, one of the familiar objects was substituted with a novel object, and the rats were reintroduced to the box. The exploration time for each object in both the training and testing phases was recorded over a 3-min period using an overhead camera. The discrimination index was calculated by comparing the difference in time spent exploring the familiar object versus the novel one, relative to the total exploration time. Additionally, the time spent exploring each object was recorded to quantify the animals’ memory performance and preference for novelty (Agrawal et al. 2020).

### Biochemical analysis

#### Histopathological examination

The brains were meticulously dissected and immersed in 10% neutral buffered formalin for 72 h to ensure proper fixation. After fixation, the tissues were subjected to sequential ethanol gradients for dehydration, followed by clearing in xylene. They were then infiltrated and embedded in Paraplast embedding medium. Using a rotary microtome, 5 μm-thick sagittal sections were obtained to specifically highlight the striatal regions in various samples. These sections were subsequently mounted on glass slides. For histological analysis, the tissue sections were stained using Hematoxylin and Eosin (H&E) (Bancroft and Gamble 2008).

#### Immunohistochemical examination of GFAP

The paraffin-embedded striatal sections were first deparaffinized and washed in PBS for 10 min, followed by incubation with 3% hydrogen peroxide for 10 min to block endogenous peroxidase activity. After rinsing with distilled water, the sections were mounted on positively charged slides and processed using the avidin–biotin-peroxidase complex (ABC) method. These slides were then incubated with a mouse monoclonal antibody against glial fibrillary acidic protein (GFAP) (Servicebio, Cat no. GB12090-100, Dilution: 1:500) to label astrocytes.

Next, the necessary reagents for the ABC method were applied, using the Vectastain ABC-HRP kit (Vector Laboratories), followed by peroxidase labeling of the antibody and visualization with diaminobenzidine (DAB) (Sigma-Aldrich, USA). To ensure specificity, negative control slides were prepared by replacing the primary or secondary antibodies with non-immune serum. The immunohistochemically stained sections were examined under an Olympus BX-53 microscope. Quantification of GFAP expression in the striatum, expressed as a percentage of the stained area, was performed using ImageJ version 1.4 (NIH, LOCI, United States).

#### ELISA assay of PGC-1α, NF-κB, TNF-α, IL-6 and SOD

The PGC-1α, NF-κB, TNF-α, IL-6 and SOD levels were assessed in striatum according to the manufacturer’s protocols (Cat. No. MBS762203), (Cat. No. MBS015549), (Cat. No. MBS282960), (Cat. No. MBS269892) and (Cat. No. MBS2707324), respectively, (MyBioSource, CA, United States). The results of PGC-1α and SOD were expressed as ng/mg protein. While the results of NF-κB, TNF-α and IL-6 were exhibited as pg/mg protein.

#### PCR evaluation of HIF-1α, GLUT1, GLUT3, HKII, BDNF, Nrf2 and caspase-3

To evaluate the mRNA expression levels of HIF-1α, GLUT1, GLUT3, HKII, BDNF, Nrf2 and Caspase-3, striatal samples were homogenized in a lysis buffer. Total RNA was then extracted using the RNeasy Mini Kit, and the RNA purity was verified via spectrophotometry at an absorbance of 260–280 nm. Complementary DNA (cDNA) synthesis followed the manufacturer's guidelines (Promega, Leiden, The Netherlands). Quantitative RT-PCR was performed to measure the expression of HIF-1α, GLUT1, GLUT3, HKII, BDNF, Nrf2 and Caspase-3, using SYBR Green Master Mix (Applied Biosystems, CA, USA) as per the provided instructions. Primer sequences for the analysis are listed in Table 1. PCR amplification was conducted over 40 cycles, with thermal cycling conditions set at 95 °C for 15 s, 60 °C for 60 s, and 72 °C for 60 s. After completing the RT-PCR process, results were analyzed using the cycle threshold (Ct) method, with expression levels presented as relative fold changes normalized against the control gene (GAPDH).

**Table 1:** PCR Primers

Gene	Primers Sequences
HIF-1α	F: 5′- CCAGATTCAAGATCAGCCAGCA-3′
	R: 5′- GCTGTCCACATCAAAGCAGTACTCA-3′
GLUT1	F: 5′- GCCTGAGACCAGTTGAAAGCAC-3′
	R: 5′- CTGCTTAGGTAAAGTTACAGGAG-3′
GLUT3	F: 5′- AACAGAAAGGAGGAAGACCA-3′
	R: 5′- CGCAGCCGAGGGGAAGAACA-3′
HKII	F: 5′- GAGAAAGCTCAGCATCGTGG-3′
	R: 5′- TCCATTTGTACTCCGTGGCT-3′
BDNF	F: 5′- AAGCTTATGACCATCCTTTTCCTTAC −3′
	R: 5′- GAATTCCTATCTTCCCCTTTTAATGG −3′
Nrf2	F: 5′- CGCCTTGAAGCTCATCTCAC −3′
	R: 5′- TTCTTGCCTCTCCTGCGTAT −3′
Caspase-3	F: 5′- CTGGTATTGAGGCAGACAGTGG −3′
	R: 5′- CAGCACCCTACACAGAGACTGAA −3′
GAPDH	F: 5′- TGGCATTGTGGAAGGGCTCA-3'
	R: 5′-TGGATGCAGGGATGATGTTCT-3'

*HIF-1α* Hypoxia-inducible factor 1-alpha, *GLUT1* Glucose transporter 1, *GLUT3* Glucose transporter 3, *HKII* Hexokinase II, *BDNF* Brain-derived neurotrophic factor, *Nrf2* nuclear factor erythroid 2-related factor 2, *GAPDH* Glyceraldehyde-3-phosphate dehydrogenase

#### Western blot analysis of phospho-PI3K p85/p55 (Tyr458, Tyr199), phospho-AKT (Ser473), phospho-CREB (Ser133) and SIRT1

Striatal protein extraction was done using radioimmunoprecipitation assay (RIPA) buffer (Bio Basic Inc, Ontario, Canada). The lysate product was incubated on ice for 30 min, furthermore, centrifugation for 30 min at 16,000xg at 4 ºC was done for the removal of cell debris. Following the extraction of proteins from the striatal tissues, the protein concentration was determined using the Bradford assay to ensure equal loading. The samples were subsequently combined with 2 × Laemmli sample buffer, and the pH was adjusted to 6.8 for optimal conditions. Protein samples were separated using 12% sodium dodecyl sulfate–polyacrylamide gel electrophoresis (SDS-PAGE), after which the proteins were transferred to polyvinylidene fluoride (PVDF) membranes utilizing a semi-dry transfer apparatus (Bio-Rad Laboratories, CA, USA). To block non-specific binding, the membranes were incubated in a tris-buffered saline with Tween 20 (TBST) buffer in addition to bovine serum albumin 3% (BSA) for 1 h at room temperature.

Next, the membranes were incubated overnight at 4 °C with primary antibodies: Phospho-PI3K p85/p55 (Tyr458, Tyr199) Polyclonal Antibody (1:1000; Cat. No. PA5-17,387), Phospho-AKT (Ser473) Polyclonal Antibody (1:2000; Cat. No. 600–401-268), Phospho-CREB (Ser133) Polyclonal Antibody (1:200, Cat No. PA1-4619), anti-SIRT1 Polyclonal Antibody (1:1000; Cat. No. PA5-17,074) and beta Actin (β-actin) Polyclonal Antibody (1:1000, Cat No. PA5-16,914) (ThermoFisher Scientific, MA, USA). After primary antibody incubation, the Goat anti-Rabbit IgG (1:10,000, Cat no. A16110 ThermoFisher Scientific, United States) secondary antibody was added and incubated at room temperature for 1 h. Densitometric analysis was performed to quantify the targeted protein utilizing the ChemiDoc™ MP Imaging System. The expression of beta-actin protein served as a reference for normalizing the results, which were reported in arbitrary units (AU).

### Statistical analysis

Statistical analyses were performed utilizing MS Excel and GraphPad Prism software (version 8.0.2). All variables were expressed as mean ± standard deviation (SD). Biochemical assay data were analyzed using one-way ANOVA, followed by Tukey's post hoc multiple-comparisons test. The open field test assessed ambulation and rearing frequencies utilizing the Kruskal–Wallis test, followed by Dunn's multiple comparisons. The novel object recognition test (NORT) evaluated exploration time differences between familiar and novel objects by two-way ANOVA. Results were considered significant at P < 0.05 for all assessments.

## Results

### Effect of Canagliflozin on behavioral tests

**Fig. 1:**
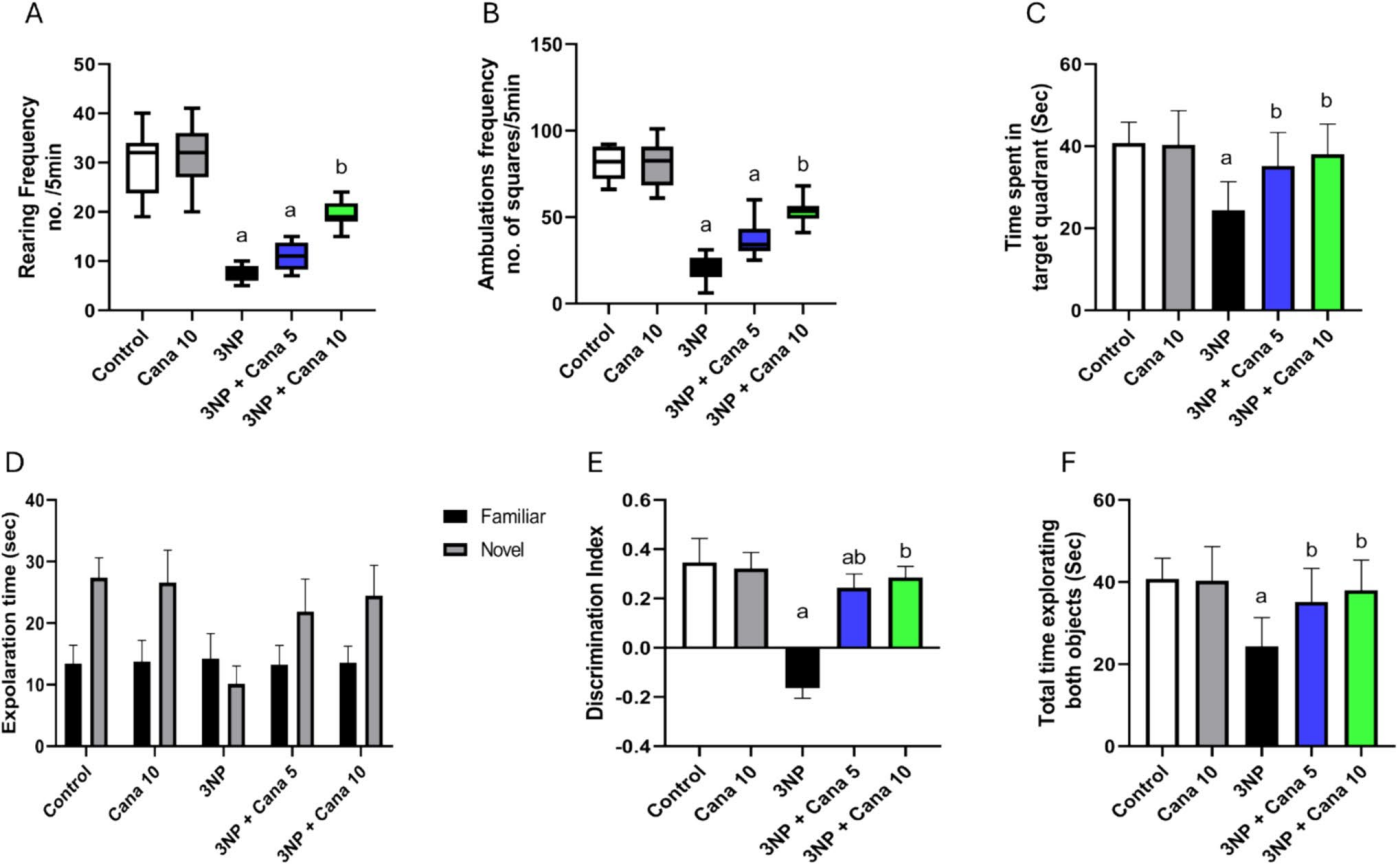
Effect of Cana on OPT, MWM and NORT against 3NP induced HD in rats. **A** rearing frequency, **B** ambulation frequency in open field test, **C** time spent in target quadrant in Morris water maze test, **D** exploration time of novel and familiar object, **E** discrimination index and **F** total time exploring both objects in the novel object recognition test. Nonparametric data (rearing and ambulation frequency) were presented as boxplots showing the median, 25th, and 75th percentiles and analyzed using the Kruskal–Wallis test followed by Dunn’s post-hoc test. Parametric data were expressed as mean ± SD (*n* = 12) and analyzed using one-way ANOVA followed by Tukey’s test. Statistical significance was considered at *p* < 0.05; (a) vs. control group and (b) vs. 3NP group. 3NP, 3-nitropropionic acid; Cana, Canagliflozin

HD models exhibit locomotor and behavioral deficits that indicate striatal atrophy and motor impairment, manifesting as abnormalities in gait and hypoactivity as well as decreased spatial learning and impaired memory function (Fig. 1).

In the open field test, the injection of 10 mg/kg 3NP for 14 days exhibited a significant decrease in rearing frequency by (*P* < 0.0001, 75.83%) and ambulation frequency by (*P* < 0.0001, 75.38%), compared to the control group. However, co-treatment with Canagliflozin 10 mg/kg for 14 days reversed the effect of 3NP, leading to increase in rearing and ambulation frequency by (*P* = 0.0072, 2.68 folds) and (*P* = 0.016, 2.65-folds), respectively, compared to the 3NP diseased group.

In the Morris’s water maze test (F_(4, 55)_ = 10.32, *P* < 0.0001), intoxication with 10 mg/kg 3NP showed a (*P* < 0.0001, 61.09%) decrease in the duration spent by the rats in the target quadrant compared to the control group. Meanwhile, co-administration of Canagliflozin at doses of 5 and 10 mg/kg for 14 days, countered the effect of 3NP. This was demonstrated by a significant increase in the time spent in the target quadrant by (*P* = 0.0052, 1.89-fold) and (*P* = 0.0002, 2.15-fold), respectively, compared to the group treated with 3NP.

In the novel object recognition test, the amount of time spent exploring novel object compared to the familiar object, time spent exploring novel object, the discrimination index and the total time spent exploring both objects were assessed. Regarding the amount of time spent exploring novel object compared to the familiar object, rats injected with 10 mg/kg 3NP displayed no significant difference (*P* = 0.4431). However, co-treatment with 5 and 10 mg/kg Cana significantly improved the time spent exploring novel object by (*P* < 0.0001, 1.65 folds) and (*P* < 0.0001, 1.8 folds), respectively, compared to the familiar object. Regarding time spent exploring novel object, administration of 10 mg/kg 3NP caused the rats to spend significantly less time by (*P* < 0.0001, 62.86%) compared to the control group. On the contrary, it significantly elevated with the co-treatment of 5 and 10 mg/kg Canagliflozin, by (*P* < 0.0001, 2.15-fold) and (*P* < 0.0001, 2.4-fold), respectively, compared to the 3NP group. Regarding the discrimination index (F _(4, 55)_ = 130.4, *P* < 0.0001), it significantly declined from 0.34 in the control group to (*P* < 0.0001, −0.16) in the group treated with 10 mg/kg 3NP. Conversely, co-administration of 5 mg/kg and 10 mg/kg Canagliflozin significantly improved discrimination index to (*P* < 0.0001, 0.24) and (*P* < 0.0001, 0.28), respectively. Regarding the total time spent exploring both objects (F _(4, 55)_ = 10.32, *P* < 0.0001). It decreased significantly by (*P* < 0.0001, 40.24%) with the administration of 10 mg/kg 3NP compared to control group. While co-treatment using 5 and 10 mg/kg Canagliflozin significantly increased total time spent exploring both objects by (*P* = 0.0052, 1.44 folds) and (*P* = 0.0002, 1.55 folds), respectively, compared to the 3NP group.

### Effect of Canagliflozin on histopathological examination

**Fig. 2:**
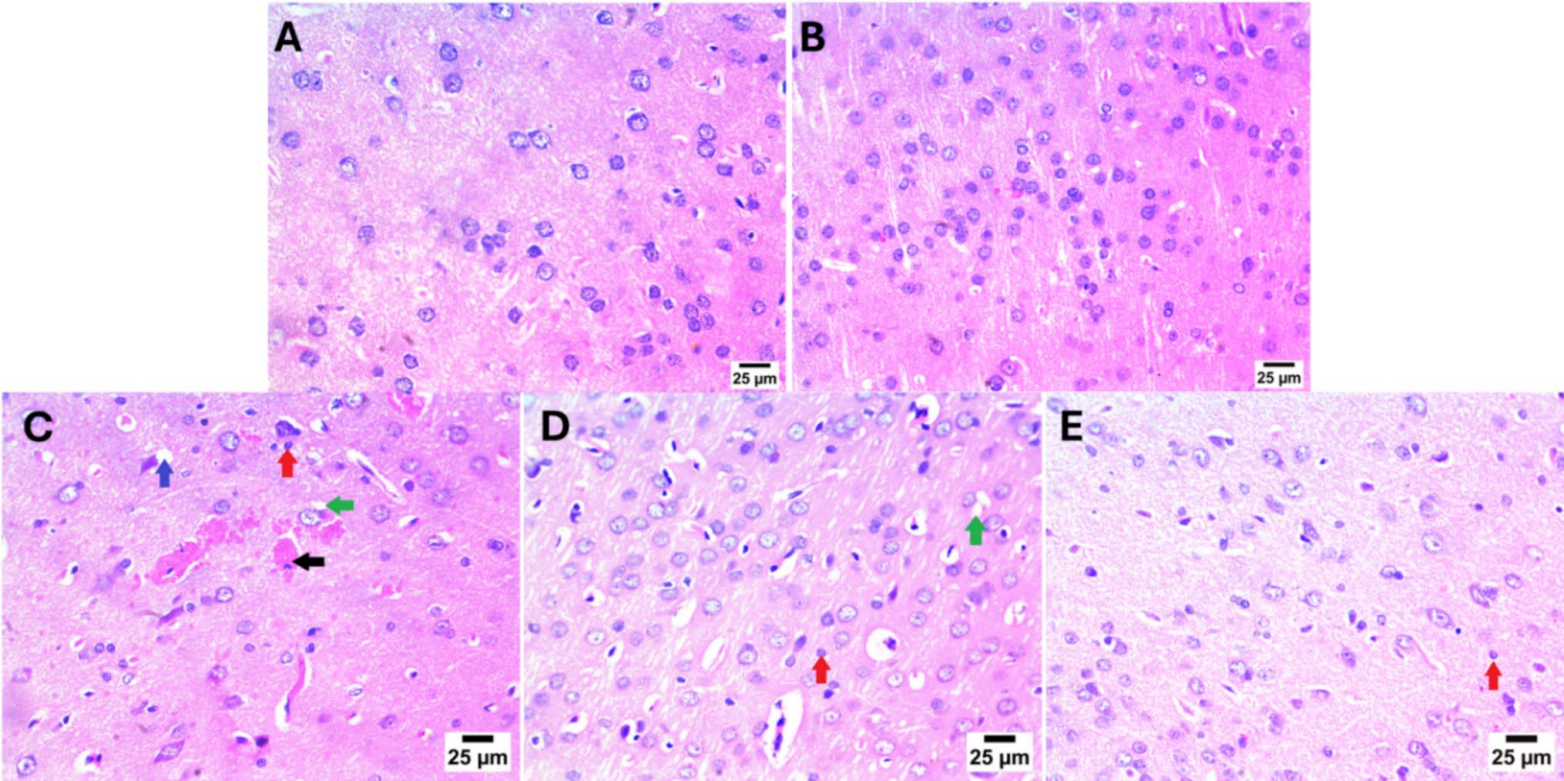
Effect of Cana on histopathological alterations against 3NP induced HD in rats. **A**–**E** photomicrographs represent staining of striatum with H&E (Scale bar 25 μm). **A** Control group, (**B**) Cana only treated group, (**C**) 3NP treated group, (**D**) Cana 5 mg/kg treated group and (**E**) Cana 10 mg/kg treated group. Perineural edema (blue arrow), perineuronal (green arrow) edema, hemorrhage (black arrow) and astrogliosis (red arrow). 3NP, 3-nitropropionic acid; Cana, Canagliflozin; H&E, hematoxylin and eosin

Striatal tissues were examined by H&E (Fig. 2). Photomicrographs of control and Canagliflozin treated only groups displayed normal histological structure of striatum with apparent intact neurons. Oppositely, intoxication with 3NP showed perineural (blue arrow) and perineuronal (green arrow) edema with hemorrhage (black arrow) and astrogliosis (red arrow). Interestingly, treatment with low dose 5 mg/kg Canagliflozin displayed mild perineuronal edema (green arrow) with moderate astrogliosis (red arrow). Similarly, treatment with higher dose 10 mg/kg Canagliflozin showed mild astrogliosis (red arrow) only.

### Effect of Canagliflozin on GFAP immunoexpression

**Fig. 3:**
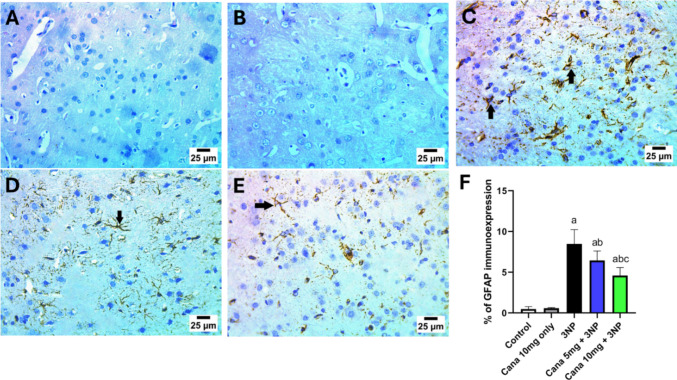
Effect of Cana on striatal GFAP immunoreactivity against 3NP induced HD in rats. **A**–**F** photomicrographs represent immunohistochemical staining of GFAP (Scale bar 25 μm). **A** Control group, (**B**) Cana only treated group, (**C**) 3NP treated group, (**D**) Cana 5 mg/kg treated group, (**E**) Cana 10 mg/kg treated group and (**F**) % area of GFAP immunoexpression. Parametric data were expressed as mean ± SD and analyzed using one-way ANOVA followed by Tukey’s test. Statistical significance was considered at *p* < 0.05; (a) vs. control group, (b) vs. 3NP group and (c) vs. 3NP + Cana 5 mg group. 3NP, 3-nitropropionic acid; Cana, Canagliflozin. GFAP; glial fibrillary acidic protein

Immunostaining was employed to evaluate the immunoreactivity of striatal GFAP, serving as a marker to determine the extent of astrocyte activation (Fig. 3). The control and Cana treated only groups showed negative reaction for GFAP in striatum. Intoxication using 10 mg/kg 3NP displayed sever positive reaction for GFAP in striatum. Oppositely, striatum part obtained from the co-administration of Canagliflozin 5 and 10 mg/kg treated group revealed moderate and mild positive reaction for GFAP in striatum (arrow), respectively. Statistical analysis displayed a significant difference between groups (F_(4, 45)_ = 114.8, *P* < 0.0001). Rats treated with 10 mg/kg 3NP caused a significant increase in immunoreactivity by (*P* < 0.0001, 17.73-fold), compared to normal group. Co-treatment with Cana 5 mg/kg significantly reduced the immunoexpression of GFAP by (*P* = 0.0007, 24.05%), compared to that seen in the diseased group. In addition, co-administration of Cana 10 mg/kg significantly decreased the immunostaining by (*P* < 0.0001, 45.85%) compared to 3NP treated mice. Moreover, treatment using high dose 10 mg/kg Cana displayed significant differences (*P* < 0.0024, 28.71%), when compared to the 5 mg/kg Cana treated group.

### Effect of Canagliflozin on the expression of HIF-1α, GLUT1, GLUT3 and HKII

**Fig. 4:**
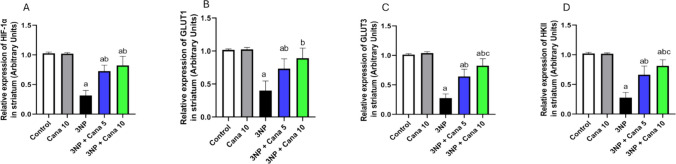
Effect of Canagliflozin on expression of HIF-1α, GLUT1, GLUT3 and HKII in striatum against 3NP induced HD in rats. **A**) HIF-1α, **B**) GLUT1, **C**) GLUT3, and **D**) HKII. Parametric data were expressed as mean ± SD (n = 7) and analyzed using one-way ANOVA followed by Tukey’s test. Statistical significance was considered at *p* < 0.05; (a) vs. control group, (b) vs. 3NP group and (c) vs. 3NP + Cana 5 mg group. 3NP, 3-nitropropionic acid; Cana, Canagliflozin, HIF-1α; hypoxia-inducible factor 1-alpha, GLUT1; glucose transporter 1, GLUT3; glucose transporter 3, HKII; hexokinase II

The expression of HIF-1α, GLUT1, GLUT3 and HKII in striatum were assessed (Fig. 4), and the statistical analysis using one-way ANOVA revealed significant differences among the experimental groups for (HIF-1α: F_(4, 30)_ = 72.13, *P* < 0.0001), (GLUT1: F_(4, 30)_ = 34.7, *P* < 0.0001), (GLUT3: F_(4, 30)_ = 99.42, *P* < 0.0001) and (HKII: F_(4, 30)_ = 83.56, *P* < 0.0001). Intoxication with 3NP caused a significant reduction in the expression of HIF-1α, GLUT1, GLUT3 and HKII to (*P* < 0.0001; 69.26%), (*P* < 0.0001; 60.67%), (*P* < 0.0001; 72.77%) and (*P* < 0.0001; 72.82%), respectively, compared to normal group. In contrast, treatment with Cana at 5 mg/kg significantly elevated the expression of HIF-1α, GLUT1, GLUT3 and HKII (*P* < 0.0001; 2.3 folds), (*P* < 0.0001; 1.83 folds), (*P* < 0.0001; 2.33 folds) and (*P* < 0.0001; 2.39 folds), respectively, compared to 3NP treated group. Similarly, treatment with Cana 10 mg/kg led to a significant upregulation of HIF-1α, GLUT1, GLUT3 and HKII (*P* < 0.0001; 2.6 folds), (*P* < 0.0001; 2.22 folds), (*P* < 0.0001; 2.99 folds) and (*P* < 0.0001; 2.93 folds), respectively, compared to 3NP treated group. In addition, treatment with Cana 10 mg/kg significantly further enhanced the expression of GLUT3 and HKII (*P* = 0.0025; 1.28 folds) and (*P* < 0.0301; 1.22 folds), respectively, when compared to Cana 5 mg/kg group.

### Effect of Canagliflozin on the expression levels of PI3K, AKT, CREB and BDNF

**Fig. 5:**
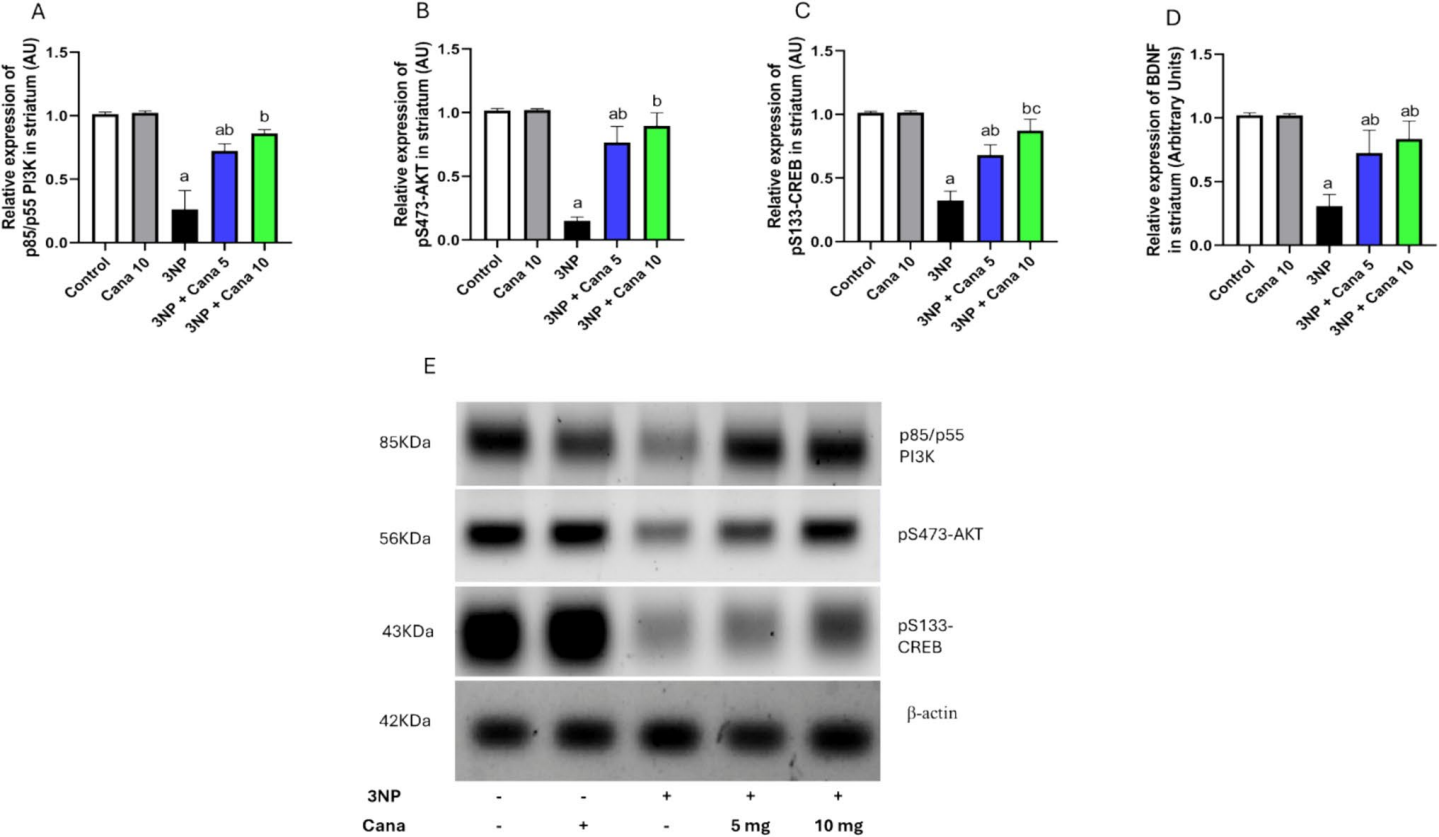
Effect of Canagliflozin on expression of p-PI3k, p-AKT, p-CREB and BDNF in striatum against 3NP induced HD in rats. **A**) p-PI3K, **B**) p-AKT, **C**) p-CREB, **D**) BDNF and **E**) Cropped western blot gels. Parametric data were expressed as mean ± SD (n = 3, except BDNF n = 7) and analyzed using one-way ANOVA followed by Tukey’s test. Statistical significance was considered at *p* < 0.05; (a) vs. control group, (b) vs. 3NP group and (c) vs. 3NP + Cana 5 mg group. 3NP, 3-nitropropionic acid; Cana, Canagliflozin, p-PI3k; phosphorylated phosphoinositide 3-kinase, p-AKT; phosphorylated protein kinase B, p-CREB; phosphorylated cAMP-responsive element-binding protein, BDNF; brain-derived neurotrophic factor

The expression levels of PI3K, AKT, CREB and BDNF in striatum were evaluated (Fig. 5), and a one-way ANOVA revealed statistically significant differences across the experimental groups for (PI3K: F_(4, 10)_ = 55.99, *P* < 0.0001), (AKT: F_(4, 10)_ = 71.43, *P* < 0.0001), (CREB: F_(4, 10)_ = 63.21, *P* < 0.0001) and (BDNF: F_(4, 30)_ = 50.37, *P* < 0.0001). Exposure to 3NP caused a marked reduction in PI3K, AKT, CREB, and BDNF levels by (*P* < 0.0001; 74.01%), (*P* < 0.0001; 85.24%), (*P* < 0.0001; 68.09%) and (*P* < 0.0001; 69.79%), respectively, when compared to control group. Oppositely, treatment with Cana 5 mg/kg attenuated 3NP effect with obvious significant elevation in the levels of PI3K, AKT, CREB and BDNF (*P* = 0.0001; 2.74 folds), (*P* < 0.0001; 5.11 folds), (*P* < 0.0001; 2.35 folds) and (*P* < 0.0001; 2.38 folds), respectively, compared to 3NP treated group. Furthermore, treatment with a higher dose of Cana 10 mg/kg resulted in significant increases in the expression of PI3K, AKT, CREB and BDNF (*P* < 0.0001; 3.26 folds), (*P* < 0.0001; 5.97 folds), (*P* < 0.0001; 2.7 folds) and (*P* < 0.0001; 2.7 folds), respectively, compared to 3NP treated group. Notably, treatment with Cana 10 mg/kg significantly stimulated the expression of CREB (*P* = 0.0249; 1.28 folds), when compared to Cana 5 mg/kg group.

### Effect of Canagliflozin on the expression of SIRT1, Nrf2, and PGC-1α

**Fig. 6:**
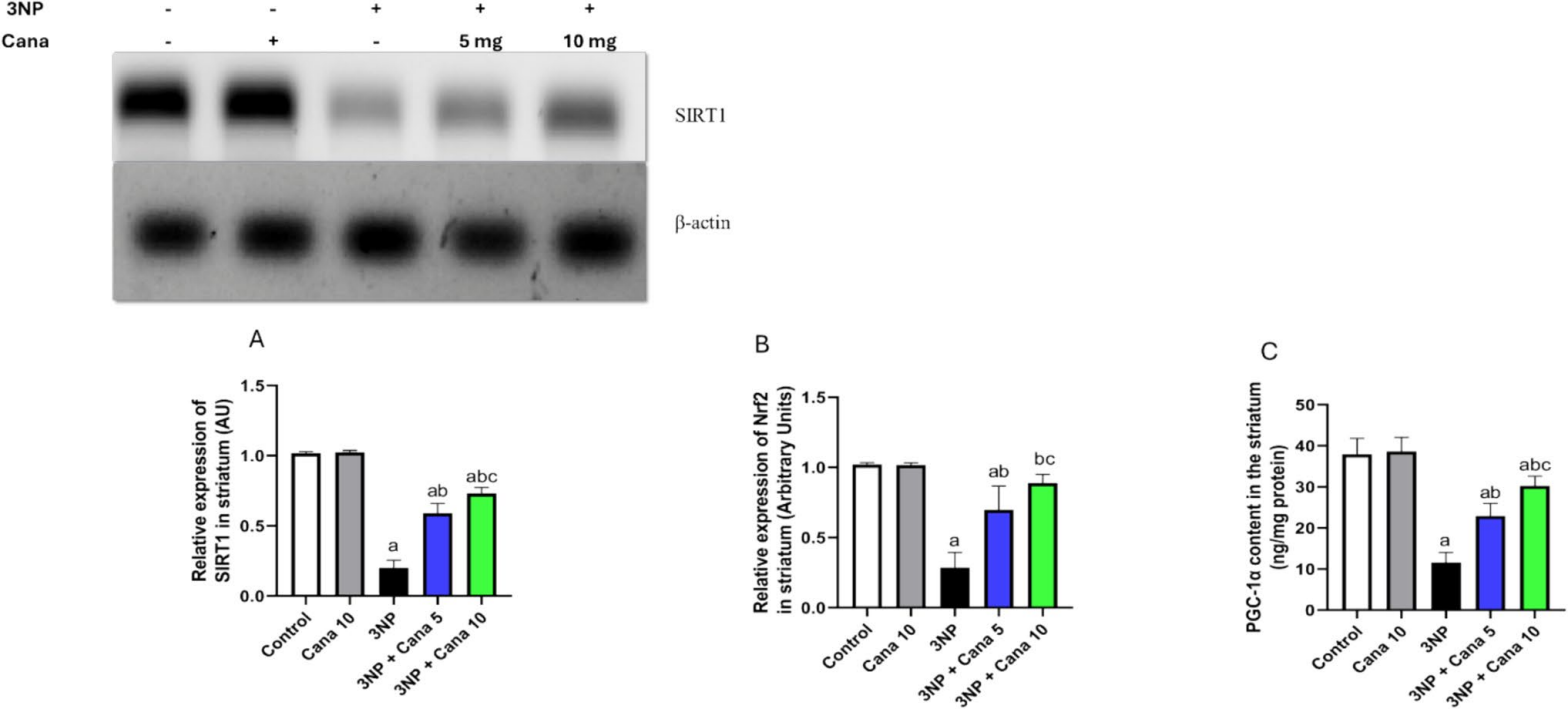
Effect of Canagliflozin on expression of SIRT1, Nrf2, and PGC-1α in striatum against 3NP induced HD in rats. **A**) SIRT1, **B**) Nrf2, and **C**) PGC-1α. Parametric data were expressed as mean ± SD (n = 7, except SIRT1 n = 3) and analyzed using one-way ANOVA followed by Tukey’s test. Statistical significance was considered at *p* < 0.05; (a) vs. control group, (b) vs. 3NP group and (c) vs. 3NP + Cana 5 mg group. 3NP, 3-nitropropionic acid; Cana, Canagliflozin, SIRT1; sirtuin 1, Nrf2; nuclear factor erythroid 2-related factor 2, PGC-1α; peroxisome proliferator-activated receptor gamma co-activator-1-alpha

The levels of SIRT1, Nrf2, and PGC-1α in the striatum were evaluated, and analyzed revealing significant differences among the groups (SIRT1: F_(4, 10)_ = 170.6, *P* < 0.0001), (Nrf2: F_(4, 30)_ = 73.55, *P* < 0.0001) and (PGC-1α: F_(4, 30)_ = 92.68, *P* < 0.0001) (Fig. 6). Exposure to 3NP caused a significant reduction in the expression of SIRT1 (*P* < 0.0001; 80.32%), Nrf2 (*P* < 0.0001; 71.98%) and PGC-1α (*P* < 0.0001; 69.46%), compared to the control group. Conversely, treatment with Canagliflozin at a dose of 5 mg/kg led to a significant upregulation in the expression of SIRT1 (*P* < 0.0001; 2.95-fold), Nrf2 (*P* < 0.0001; 2.44-fold) and PGC-1α (*P* < 0.0001; 1.97-fold), compared to 3NP treated group. Similarly, the higher dose of Canagliflozin 10 mg/kg produced even more significant enhancement in the levels of SIRT1 (*P* < 0.0001; 3.65-fold), Nrf2 (*P* < 0.0001; 3.11-fold) and PGC-1α (*P* < 0.0001; 2.6 -fold), compared to 3NP treated group. In addition, treatment with Cana 10 mg significantly boosted the expression of SIRT1, Nrf2 and PGC-1α (*P* = 0.023; 1.23 folds), (*P* = 0.0058; 1.27 folds) and (*P* = 0.0011; 1.32 folds), respectively, when compared to Cana 5 mg/kg group.

### Effect of Canagliflozin on the expression of NF-κB, TNF-α, IL-6, caspase-3 and SOD

**Fig. 7:**
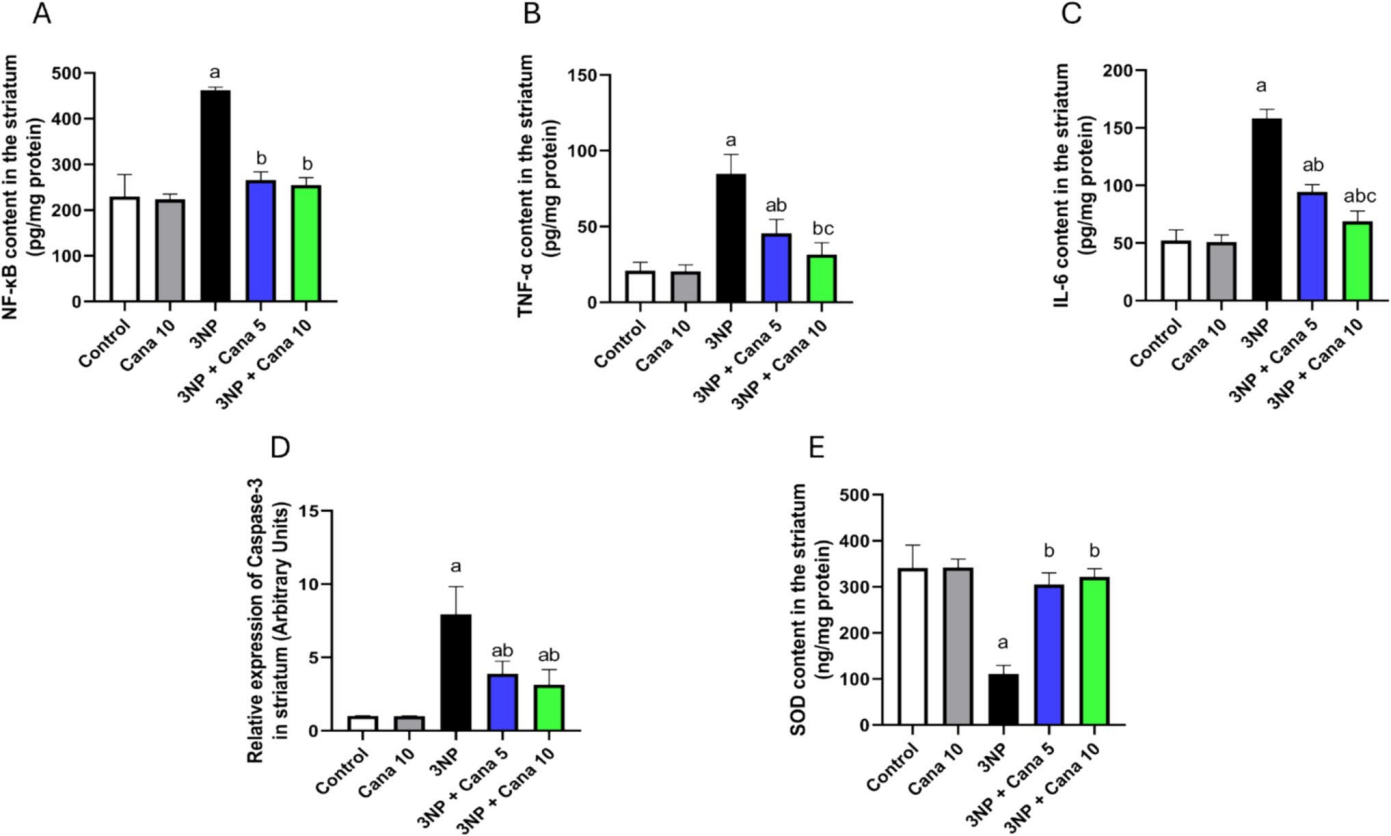
Effect of Canagliflozin on expression of NF-κB, TNF-α, IL-6, caspase-3 and SOD in striatum against 3NP induced HD in rats. **A**) NF-κB, **B**) TNF-α, **C**) IL-6, **D**) caspase-3 and **E**) SOD. Parametric data were expressed as mean ± SD (*n* = 7) and analyzed using one-way ANOVA followed by Tukey’s test. Statistical significance was considered at *p* < 0.05; (a) vs. control group, (b) vs. 3NP group and (c) vs. 3NP + Cana 5 mg group. 3NP, 3-nitropropionic acid; Cana, Canagliflozin, NF-κB; Nuclear factor kappa B, TNF-α; Tumor Necrosis Factor alpha, IL-6; Interleukin 6, SOD; superoxide dismutase

Statistical analysis displayed a significant difference in the levels of NF-κB, TNF-α, IL-6, caspase-3 and SOD, (NF-κB: F_(4, 30)_ = 115, *P* < 0.0001), (TNF-α: F_(4, 30)_ = 69.28, *P* < 0.0001), (IL-6: F_(4, 30)_ = 226.6, *P* < 0.0001), (caspase-3: F_(4, 30)_ = 52.83, *P* < 0.0001) and (SOD: F_(4, 30)_ = 82.26, *P* < 0.0001) (Fig. 7). Administration of 3NP resulted in a significant increase in NF-κB (*P* < 0.0001; twofold), TNF-α (*P* < 0.0001; 4.07-fold), IL-6 (*P* < 0.0001; 3.02-fold), and caspase-3 (*P* < 0.0001; 7.81-fold), when compared to the control group. However, 3NP displayed a significant reduction in the level of SOD (*P* < 0.0001; 67.45%), in comparison with normal group. Oppositely, treatment with Cana 5 mg/kg significantly decreased the levels of NF-κB (*P* < 0.0001; 42.39%), TNF-α (*P* < 0.0001; 46.29%), IL-6 (*P* < 0.0001; 40.25%), and caspase-3 (*P* < 0.0001; 50.97%), compared to 3NP treated group. Similarly, treatment with Cana 10 mg/kg significantly downregulated the expression of NF-κB (*P* < 0.0001; 44.91%), TNF-α (*P* < 0.0001; 62.97%), IL-6 (*P* < 0.0001; 56.41%), and caspase-3 (*P* < 0.0001; 60.5%), compared to 3NP treated group. Regarding SOD, treatment with Cana 5 mg/kg and 10 mg/kg displayed a significant increase in the levels of SOD (*P* < 0.0001; 2.74-fold) and (*P* < 0.0001; 2.9-fold), respectively, compared to 3NP group. In addition, treatment with Cana 10 mg/kg significantly reduced the expression of TNF-α (*P* = 0.0307; 31.05%), and IL-6 (*P* < 0.0001; 27.05%), when compared to Cana 5 mg/kg group.

## Discussion

Huntington’s disease (HD) is a rare, dominantly inherited neurodegenerative disorder, primarily characterized by progressive degeneration of the striatum and cortex, leading to motor, cognitive, and behavioral impairments (Tabrizi et al. 2020; McColgan and Tabrizi 2018). Additionally, HD is linked to impairments in brain oxygenation, energy metabolism, and mitochondrial function (Mosconi 2013; Sato and Morishita 2015). Mitochondrial dysfunction plays a central role by disrupting redox balance and hindering oxidative phosphorylation, which contributes to hypoxia and accelerates neurodegenerative processes (Browne 2008).

**Scheme 2:**
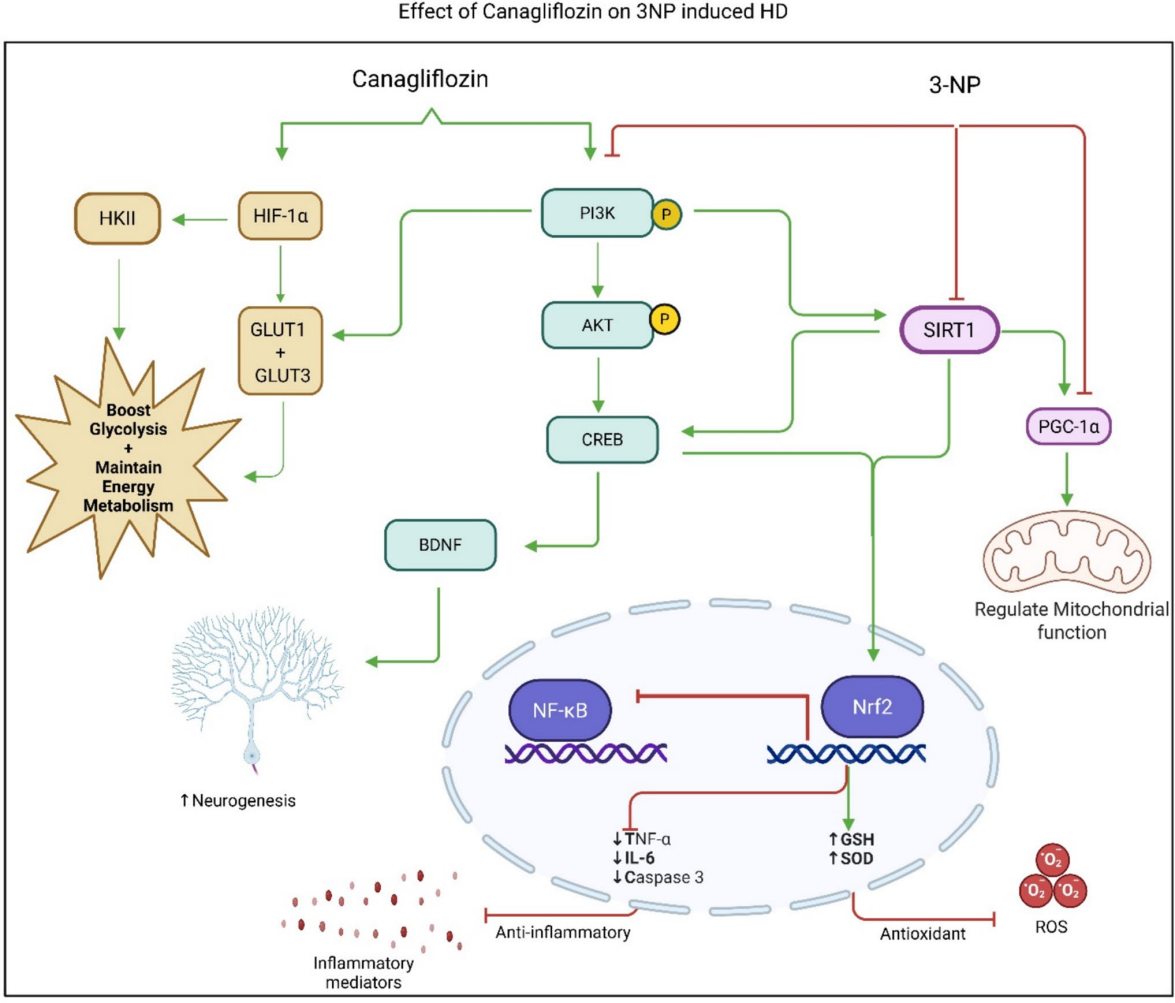
Proposed mechanistic pathway illustrating the effects of Canagliflozin on 3NP induced HD

This study emphasizes the neuroprotective effects of canagliflozin, an SGLT2 inhibitor, in mitigating 3NP-induced neurotoxicity in Huntington’s disease model. Canagliflozin exhibits several protective mechanisms: (1) it alleviates cognitive and motor deficits while also improving histological and immunohistopathological conditions; (2) it enhances mitochondrial function and energy metabolism by activating the HIF-1α/GLUT1/GLUT3 pathway; (3) it promotes the neuroprotective PI3K/AKT/SIRT1 signaling pathway; (4) it fosters neurogenesis through the CREB/BDNF/PGC1-α axis; and (5) it reduces oxidative stress and inflammatory mediators (Scheme 2).

The striatum, an essential area of the basal ganglia, is crucial in the regulation of motor coordination. Experimental studies administering 3NP have shown that it induces striatal lesions, leading to motor deficits, cognitive decline, and impaired memory retention (Jang and Cho 2016; D’Egidio et al. 2023). Furthermore, 3NP has been demonstrated to induce damage to pyramidal neurons in the CA1 and CA3 regions of the hippocampus, which are essential for cognitive function (Danduga et al. 2018). The current study utilized 3NP (10 mg/kg/day for 14 days) to establish a model of HD, which resulted in significant behavioral impairments, neurochemical alterations, and pathological changes, ultimately leading to cognitive deficits and memory loss.

In this study, the novel object recognition test (NORT), open field test, and Morris water maze were utilized to assess motor, behavioral, and cognitive impairments. Following 3NP administration, there was a significant reduction in the time spent exploring the novel object, total exploration time for both objects, and the discrimination index in NORT. Similarly, 3NP significantly decreased rearing and ambulation frequencies in the open field test, and the total time spent in the target quadrant in the Morris water maze. Notably, co-administration of Canagliflozin resulted in a significant improvement in locomotor activity, memory retention, spatial learning, and cognitive function compared to the 3NP-treated group, with the highest dose (10 mg/kg) showing the most pronounced effects. These findings suggest a beneficial effect of Canagliflozin on cognitive, memory, and behavioral outcomes, consistent with previous research (Arafa et al. 2017).

Histopathological evaluation of the striatum in rats injected with 3NP showed marked perineuronal edema and astrogliosis. Immunohistochemical analysis further confirmed significant GFAP expression in the 3NP-treated group, indicating increased astrogliosis. However, these pathological and immunohistochemical alterations were significantly alleviated in rats treated with Canagliflozin (5 mg/kg/day and 10 mg/kg/day for 14 days).

Impaired oxygen and glucose delivery are pathogenic mechanisms frequently encountered in neurodegenerative disorders (Correia and Moreira 2010; Gironi et al. 2011). A lack of these vital substrates disturbs the energy equilibrium in motor neurons, ultimately resulting in neuronal death. Hypoxia-inducible factor-1 is a vital transcription factor that is essential for cellular and tissue adaptation to hypoxic settings by modulating genes that enhance cell survival during low oxygen stress (Taylor and Scholz 2022). HIF-1 significantly inhibits neuronal death and facilitates neuronal differentiation in diseases like HD and PD (Zhang et al. 2011).

The activation of HIF-1α initiates the production of numerous target genes that are critical for fundamental physiological activities, such as energy and glucose metabolism, cell survival, and angiogenesis. This also enhances the redox environment while improving oxygen and glucose transport to tissues (Zhang et al. 2011). Specifically, HIF-1α stimulates the expression of glycolytic enzymes and glucose transporters, such as GLUT1 and GLUT3, which boost glycolysis, maintain normal brain energy metabolism, and mitigate oxidative stress (Wu et al. 2021; Reich and Hölscher 2022). In Alzheimer’s disease, HIF-1α has been shown to regulate tau protein phosphorylation by activating GLUT1 (Mitroshina and Vedunova 2024). Moreover, HIF-1α enhances glycolytic metabolism by upregulating essential enzymes such as HKII and enolase 1 (ENO1), hence accelerating pyruvate and ATP synthesis (Taylor and Scholz 2022; Ishida et al. 2020). Besides its metabolic advantages, HIF-1α has an anti-inflammatory effect by diminishing the synthesis of pro-inflammatory cytokines, including Interleukin 6 (IL-6) and Tumor Necrosis Factor alpha (TNF-α), while enhancing the expression of the anti-inflammatory cytokine IL-10 (Gurung et al. 2015). This complex involvement establishes HIF-1α as an essential regulator in protecting neurons and preserving cellular homeostasis in neurodegenerative disorders.

GLUT1 and GLUT3 isoforms are crucial for meeting the brain’s energy demands, with GLUT1 facilitating glucose transfer from the bloodstream into the brain’s interstitial fluid and GLUT3 mediating glucose transport directly into high-energy-demanding neurons (Muraleedharan and Dasgupta 2022). A deficiency in these glucose transporters has been implicated in a wide array of neurodevelopmental disorders, including dyslexia (Roeske et al. 2011), autism spectrum disorder (ASD) (Lesch et al. 2011), epilepsy, attention-deficit/hyperactivity disorder (ADHD) and Huntington's disease (HD) (Peng et al. 2021). Furthermore, the reduced expression of GLUT1 and GLUT3 has been linked to HD pathogenesis, as it contributes to disruptions in energy metabolism and glucose hypometabolism (Squitieri and Ciarmiello 2010; Adanyeguh et al. 2015; Li et al. 2012). The decrease in GLUT1 and GLUT3 levels results in impaired glucose uptake in the brain, particularly by neurons, leading to diminished glycolysis, and mitochondrial dysfunction, which impairs ATP synthesis (Adanyeguh et al. 2015; Russo et al. 2013; Powers et al. 2007). This disruption in glucose metabolism is a critical factor contributing to neuronal death in HD, further exacerbating disease progression (McClory et al. 2014; Iuliano et al. 2021).

Hexokinase II (HKII), an isoform regulated by hypoxia in the brain, is crucial for modulating neuronal survival, especially under diverse metabolic settings (Mergenthaler et al. 2012). HKII activity has demonstrated neuroprotection against cell death caused by hypoxia and oxidative stress (Mergenthaler et al. 2012; Gimenez-Cassina et al. 2009). It facilitates the initial phase of glycolysis by transforming glucose into glucose-6-phosphate (G6P), an essential substrate for glycolysis and the pentose phosphate pathway, so establishing HKII as a pivotal regulator of energy metabolism and redox equilibrium (Mc Cluskey 2021). Reduced expression of HKII impedes glucose metabolism, exacerbates mitochondrial dysfunction, and increases ROS production (Han et al. 2021). Furthermore, HKII ablation has been linked to heightened inflammatory responses and worse brain damage (Hu et al. 2022). Thus, the induction of HIF-1α, along with GLUT1, GLUT3, and HKII, may confer significant cytoprotective benefits by boosting energy production and contributing to cellular redox homeostasis, ultimately supporting neuronal survival in conditions like HD.

In our study, intoxication with 3NP for 14 days caused defects in energy metabolism and glucose hypometabolism which could be attributed to the marked reduction in the levels of HIF-1α, GLUT1, GLUT3, and HKII. These results are consistent with the prior published studies (Morea et al. 2017; Yang et al. 2005; Mc Cluskey 2021; Niatsetskaya et al. 2010). Oppositely, treatment with Canagliflozin has successfully abolished energy metabolism disruption by the enhancement of HIF-1α, GLUT1, GLUT3, and HKII expression. In line with our results, it was demonstrated that Empagliflozin (another SGLT inhibitor) could adjust impaired energy metabolism by increasing levels of HIF-1α and GLUT1 (Trum et al. 2020). However, the increase of GLUT3 and HKII could be related to the stimulation of the up regulatory HIF-1α (Mitroshina and Vedunova 2024; Mobasheri et al. 2005).

The PI3K/AKT/CREB/BDNF signaling pathway is essential for enhancing neuronal survival. Research indicates that brain cell proliferation is significantly dependent on this route (Zhou et al. 2020). In contrast, Dysregulated PI3K/AKT signaling in neurons results in adverse outcomes, such as elevated reactive oxygen species (ROS), mitochondrial membrane depolarization, mitochondrial fragmentation, and diminished oxidative phosphorylation and ATP production (Kim et al. 2016). Upon activation, PI3K recruits AKT. As a downstream mediator, AKT governs cell survival by phosphorylating many signaling pathways, which then leads to the production of CREB (Zhang et al. 2018).

CREB, a nuclear transcription factor, is predominantly expressed in the brain, especially in neurons, and is integral to numerous brain illnesses, including neurodegenerative and cognitive disorders. Dysregulation of CREB signaling has been associated with illnesses such as Alzheimer’s disease and Huntington’s disease, underscoring its significance in synaptic function (Amidfar et al. 2020; Saura and Cardinaux 2017).

The survival of neurons is closely linked to the upregulation of phosphorylated CREB (Nakajima et al. 2002). CREB activation initiates the transcription of genes associated with neuroprotection, memory, learning, synaptic transmission, cell survival, neuronal differentiation, and axonal growth (Freitas et al. 2013). Studies have demonstrated that CREB knockout mice die shortly after birth, indicating its essential role in development (Huang et al. 2015a). Furthermore, the PI3K/AKT pathway positively regulates CREB in striatal neurons, and this signaling cascade has been shown to protect against neurological impairment and tissue injury following cerebral ischemia (Wang et al. 2016; Zhang et al. 2013).

Moreover, the activation of CREB drives the transcription of critical proteins for synaptic function, most notably BDNF and Nrf2. BDNF is essential for synaptic transmission and long-term potentiation; its decrease is associated with neuronal atrophy and memory impairment in HD (Speidell et al. 2023). Modulating CREB levels to enhance BDNF expression may safeguard against memory impairments and cognitive decline (Amidfar et al. 2020). Furthermore, the elevation in BDNF levels mediated by the PI3K/AKT/CREB pathway enhances the synthesis of synaptic proteins, potentially mitigating symptoms of neurodegenerative disorders like HD (Mustafa et al. 2021).

The PI3K/AKT pathway facilitates glucose uptake and energy metabolism by modulating the expression of the GLUT3 transporter, hence enhancing local glucose utilization to meet the brain's energy requirements (Agostini et al. 2016; McNay et al. 2010). In this context, PI3K, AKT, CREB, and BDNF are considered critical neuro-regeneration markers and therapeutic targets because of their involvement in facilitating neurogenesis and neuronal survival (Yoo et al. 2017).

SIRT1, another crucial downstream effector of the PI3K/AKT pathway, plays a significant role in the regulation of oxidative stress, inflammation, and apoptosis (Yang et al. 2021; Ren et al. 2019). Prior studies indicated that inhibition of SIRT1 is implicated in neuroinflammation, apoptosis as well as oxidative stress (Huang et al. 2015b). In HD, SIRT1 activation alleviates neuronal cytotoxicity caused by mutant huntingtin (mHtt), but its suppression intensifies mHtt-associated toxicity. SIRT1 controls neuroinflammation by suppressing astrocyte activation, resulting in decreased levels of GFAP, a recognized hallmark of astrogliosis in HD (Shaheen et al. 2021; Korpela et al. 2024). Additionally, SIRT1 has been shown to suppress microglial activation, further reducing inflammatory damage in the brain (Li et al. 2020). SIRT1 also exerts neuroprotective effects through the deacetylation and activation of TORC1 (CREB-regulated transcription coactivator 1), which then binds to CREB to promote the transcription of BDNF, an essential neuroprotective factor in HD (Jeong et al. 2012; Zuccato and Cattaneo 2007). This coordination between PI3K/AKT, CREB, BDNF, and SIRT1 underlines the therapeutic potential of targeting these pathways in neurodegenerative diseases like HD.

Furthermore, SIRT1 is crucial in modulating gluconeogenic and glycolytic pathways via its association with the transcriptional co-activator PGC-1α. This modulation improves mitochondrial mass and functionality (Manjula et al. 2021). PGC-1α is an essential regulator of mitochondrial dynamics, since it sustains energy homeostasis, metabolism, and mitochondrial function. (Finck and Kelly 2006). Recent studies highlight its involvement in the progression and neurodegeneration seen in HD (McGill and Beal 2006). In which, metabolic dysfunction and oxidative stress appear to be linked to the impaired activity of PGC-1α caused by mutant huntingtin (mHtt) (Tabrizi et al. 2020).

The HD brain shows reduced expression of PGC-1α target genes, and its dysfunction manifests in phenotypes such as hyperactivity, limb clasping, and striatal neurodegeneration (Lin et al. 2004; Weydt et al. 2006). Increased PGC-1α activity has been demonstrated to enhance the expression of Nrf2 that protects neurons from oxidative damage generated by ROS (Gureev et al. 2019). Furthermore, it improves mitochondrial respiratory capacity, maintains ATP levels, and prolongs cellular longevity (Zhou et al. 2018). The stimulation of PGC-1α via SIRT1 and CREB phosphorylation may lead to notable behavioral enhancements and provide molecular advantages, such as the decrease of mitochondrial ROS (Lee et al. 2018).

In the present study, administration of (10 mg/kg) 3NP caused neurodegeneration by the inhibition of PI3K/AKT/CREB/BDNF pathway as well as SIRT1/PGC-1α cascade, which is in line with previous study (Mustafa et al. 2021; Cui et al. 2022). However, treatment with Canagliflozin (SGLT2 inhibitor) showed a neuroprotective effect through the enhancement of PI3K/AKT/CREB/BDNF and its downstream trajectory SIRT1/PGC-1α. This is consistent with a prior study which stated the ability of SGLT2 inhibitor (Dapagliflozin) to stimulate PI3K/AKT/CREB pathway in treatment of neurodegenerative diseases, as well as ability of Canagliflozin to activate PI3K/AKT (Huang et al. 2024; Arab et al. 2021; El-Safty et al. 2022). Moreover, Kamies et al., stated the ability of Canagliflozin to stimulate SIRT1 and BDNF to preform neuroprotection against Alzheimer’s disease (Khamies et al. 2024).

SIRT1 has been shown to activate the Nrf2 pathway, which plays a crucial role in counteracting oxidative stress (Huang et al. 2015b). Nrf2 is a transcription factor that acts as apivotal defense mechanism in neurons and glial cells through modulating the expression of numerous antioxidant enzymes, phase I and II detoxifying enzymes, and anti-inflammatory mediators. The dysregulation of Nrf2 has been associated with numerous disorders related to oxidative stress, including HD (Deshmukh et al. 2017). In HD, Nrf2 mitigates oxidative stress by activating antioxidant enzymes such as superoxide dismutase (SOD) and glutathione (GSH) (Liu et al. 2021). Furthermore, it diminishes neuroinflammation induced by the redox-sensitive transcription factor NF-κB, especially in 3NP models (Brandes and Gray 2020). Nrf2 mitigates apoptosis by suppressing caspase-3 and increasing DNA repair pathways (Kobayashi et al. 2016; Ushida and Talalay 2013). Nrf2 also has a crucial role in inhibiting pro-inflammatory cytokines, including TNF-α and IL-6 (Fragoulis et al. 2012).

On the other hand, Nuclear factor kappa B (NF-κB) is crucial to inflammation, immunological responses, and the regulation of cell proliferation and death (Li et al. 2016). Dysregulation of the NF-κB pathway facilitates the transcription of pro-inflammatory genes (e.g., cytokines, chemokines, and adhesion molecules), which are associated with HD. Under normal conditions, NF-κB remains inactive in the cytoplasm due to its association with the inhibitor protein IκB. Upon activation, IκB is phosphorylated by IκB kinase (IKK), allowing the p50 and p65 (RelA) subunits of NF-κB to translocate into the nucleus. They attach to specific DNA sequences, initiating the synthesis of mRNA associated with inflammatory and apoptotic responses, including the creation of TNF-α, IL-6, and caspase-3 (Li et al. 2016; Mattson and Camandola 2001). Inhibition of NF-κB has shown neuroprotective effects in 3NP models of HD (Napolitano et al. 2008). This inhibition is likely facilitated by the activation of the PI3K/AKT pathway and the upregulation of Nrf2 (Brandes and Gray 2020; Li et al. 2015).

This finely tuned interplay between SIRT1, Nrf2, and NF-κB presents a promising therapeutic avenue for neurodegenerative diseases such as HD, offering insights into how oxidative stress, inflammation, and cell death can be controlled through molecular signaling pathways.

Reactive oxygen species (ROS) are often generated due to mitochondrial dysfunction, contributing to enhanced oxidative stress, reduction of antioxidant defenses and elevates levels of proinflammatory cytokines, such as IL-6 and TNF-α, which can exacerbate brain injury and the progression of neurodegenerative diseases like HD (Alshehri and Imam 2021; Kwon and Koh 2020). Among these inflammatory mediators, TNF-α plays a key role in aggravating inflammation by activating NF-κB, which in turn stimulates the production of proinflammatory molecule IL-6 (Knobloch et al. 2010; Zhu et al. 2011). Furthermore, TNF-α has been shown to activate apoptosis-related proteins such caspase-3, which results in DNA fragmentation and cell death (Abdelsameea and Kabil 2018). The control of inflammatory mediators such as TNF-α and IL-6, coupled with the lowering of caspase-3 levels to prevent neuronal apoptosis, provides substantial neuroprotective benefits (Abdelsameea and Kabil 2018).

In the present study, 3NP intoxication led to a notable reduction in Nrf2 and the antioxidant enzyme SOD, accompanied by an increase in inflammatory mediators such as NF-κB, IL-6, and TNF-α, as well as the apoptotic factor caspase-3. These findings align with previous research (Jambi et al. 2024; Kang et al. 2018). In contrast, treatment with Canagliflozin (5 mg/kg and 10 mg/kg) significantly elevated Nrf2 and SOD levels, while reducing NF-κB, IL-6, TNF-α, and caspase-3. These results support earlier studies that demonstrated Canagliflozin’s potential to protect neurons by inhibiting TNF-α, IL-6, and caspase-3, while boosting SOD levels (Abdelsameea and Kabil 2018; Hassanein et al. 2023). Additionally, Ali et al., highlighted Canagliflozin’s capacity to enhance Nrf2 expression and suppress NF-κB activation, further corroborating its neuroprotective role (Ali et al. 2023).

The present study investigated the possible ability of Canagliflozin to improve the cognitive and locomotor impairment as well as the dysregulation of glucose metabolism associated with HD. Treatment with Canagliflozin could improve oxygen and glucose supply, the mitochondrial function and regulate glucose metabolism through the stimulation of HIF-1α, GLUT1, GLUT3, and HKII. Moreover, Canagliflozin could provide neuroprotective effect through the activation of PI3K/AKT/CREB/BDNF trajectory and its downstream pathway SIRT1/PGC-1α. In addition, its ability to decrease inflammatory mediators, apoptosis markers and enhancement of the antioxidant defensive system. Therefore, Canagliflozin could be a promising neuroprotective and therapeutic approach as a disease-modifying agent for HD.

## Supplementary Information

Below is the link to the electronic supplementary material.

ESM 1 (DOCX 2.66 MB)

## Data Availability

All source data for this work (or generated in this study) are available upon reasonable request.
