# Supplementary material for: Neuroprotection by canagliflozin in a Huntington’s disease model: role of HIF-1α and PI3K/AKT signaling
Source: Naunyn Schmiedebergs Arch Pharmacol. 2025 Dec 26;399(6):8245–62. doi: 10.1007/s00210-025-04891-5 (PMC13086672; doi:10.1007/s00210-025-04891-5)

**Western blot Uncropped Gels**


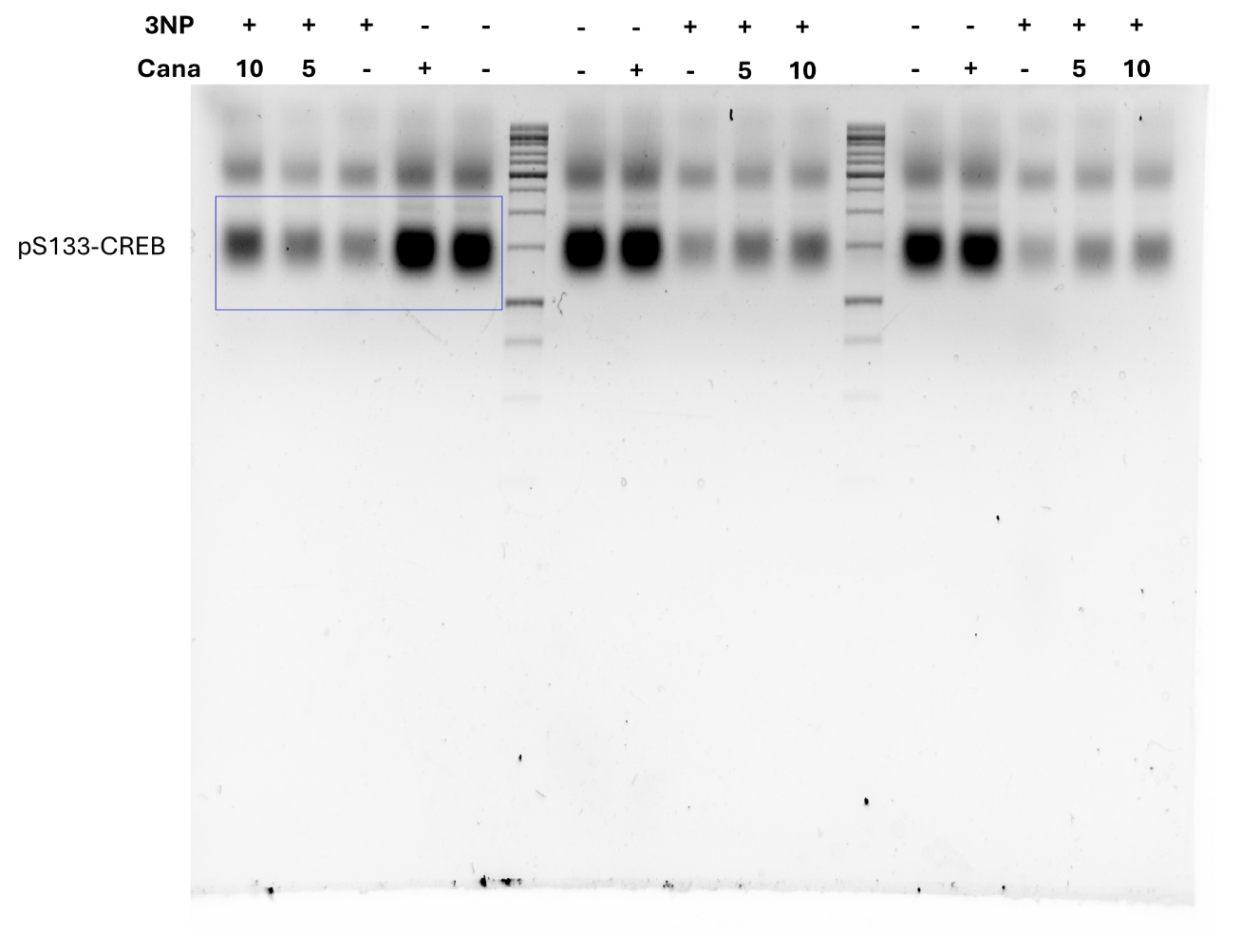


Figure 1 pS133-CREB western blot


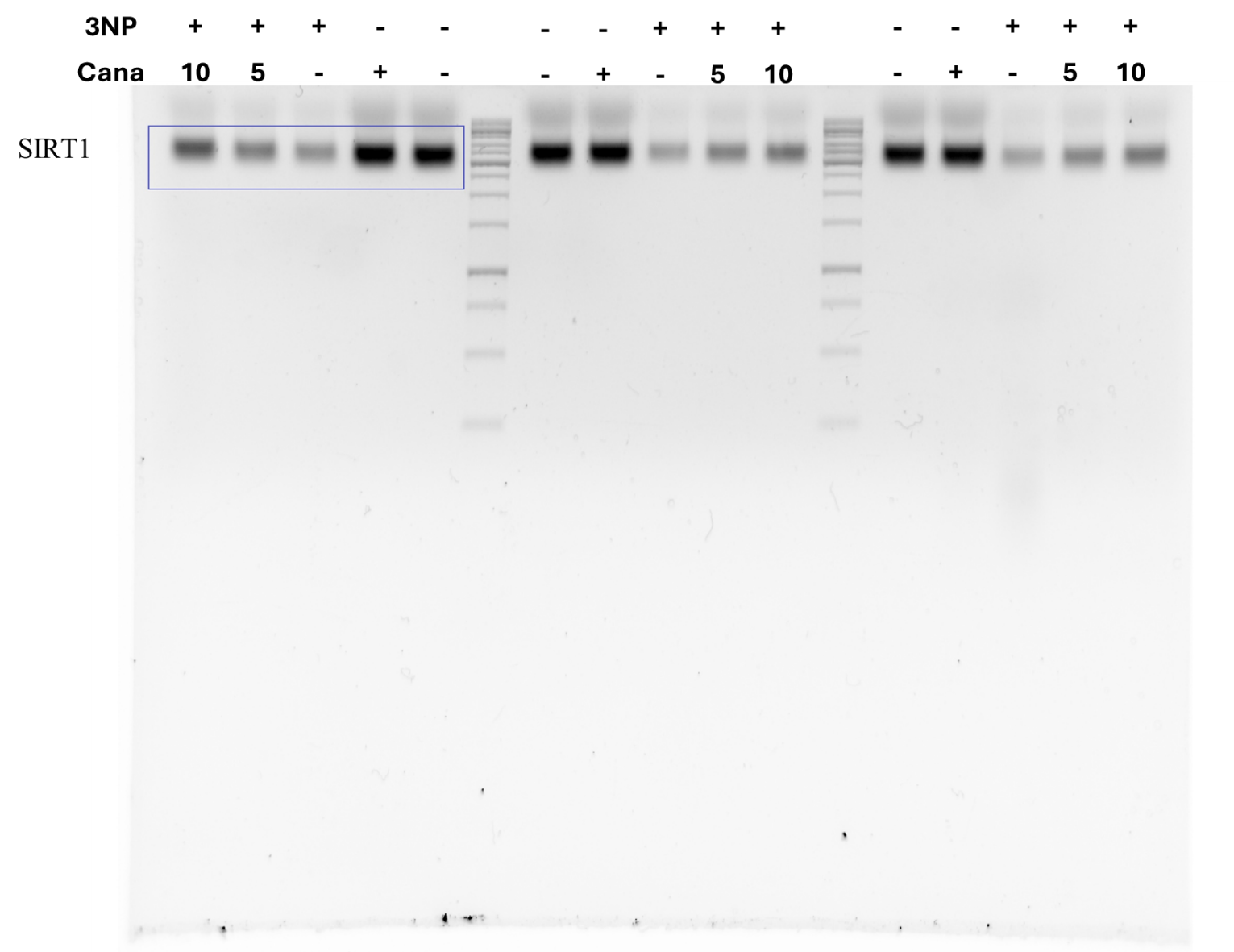


Figure 2 SIRT1 western blot


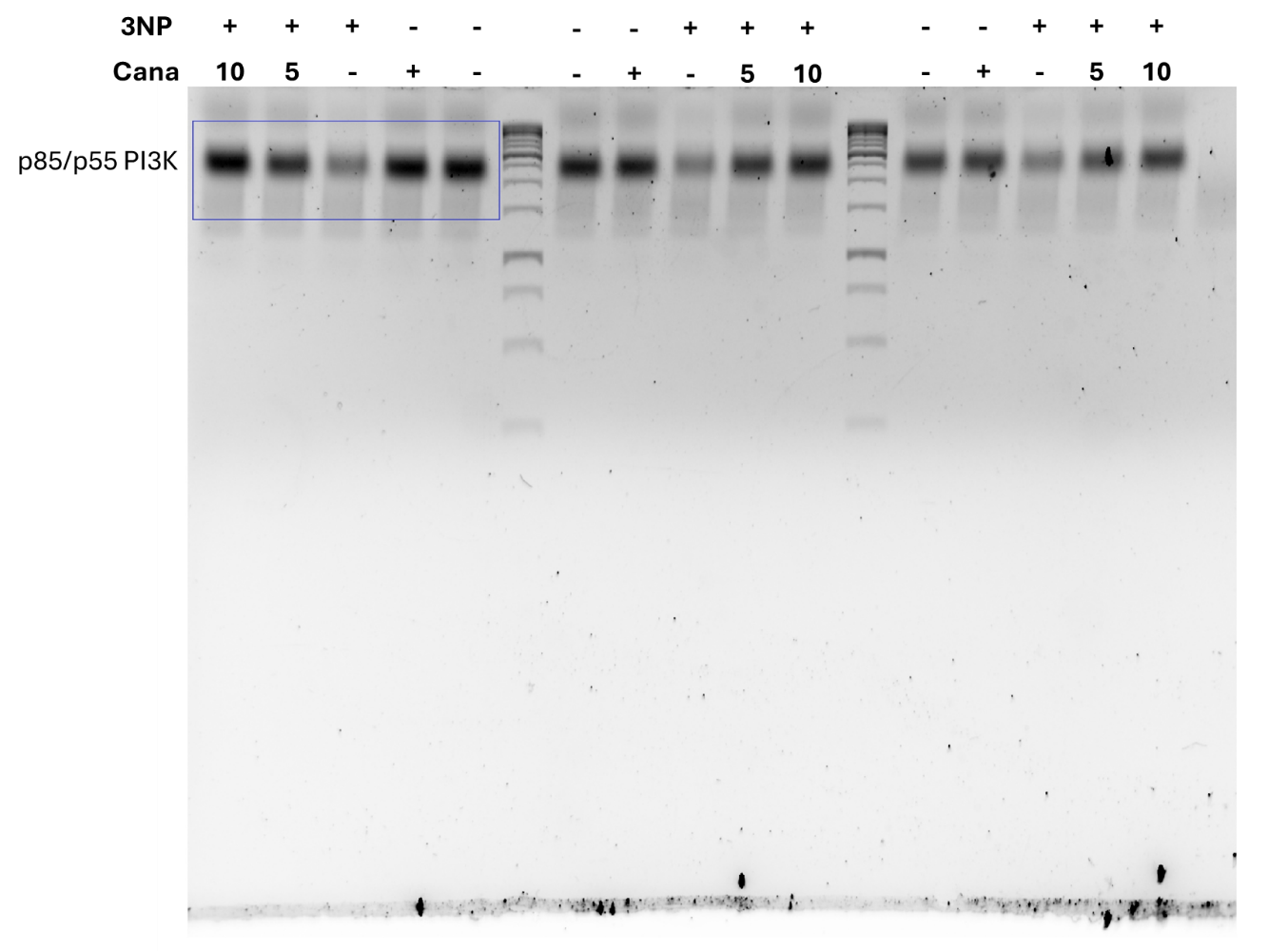


Figure 3 p85/p55 PI3K western blot


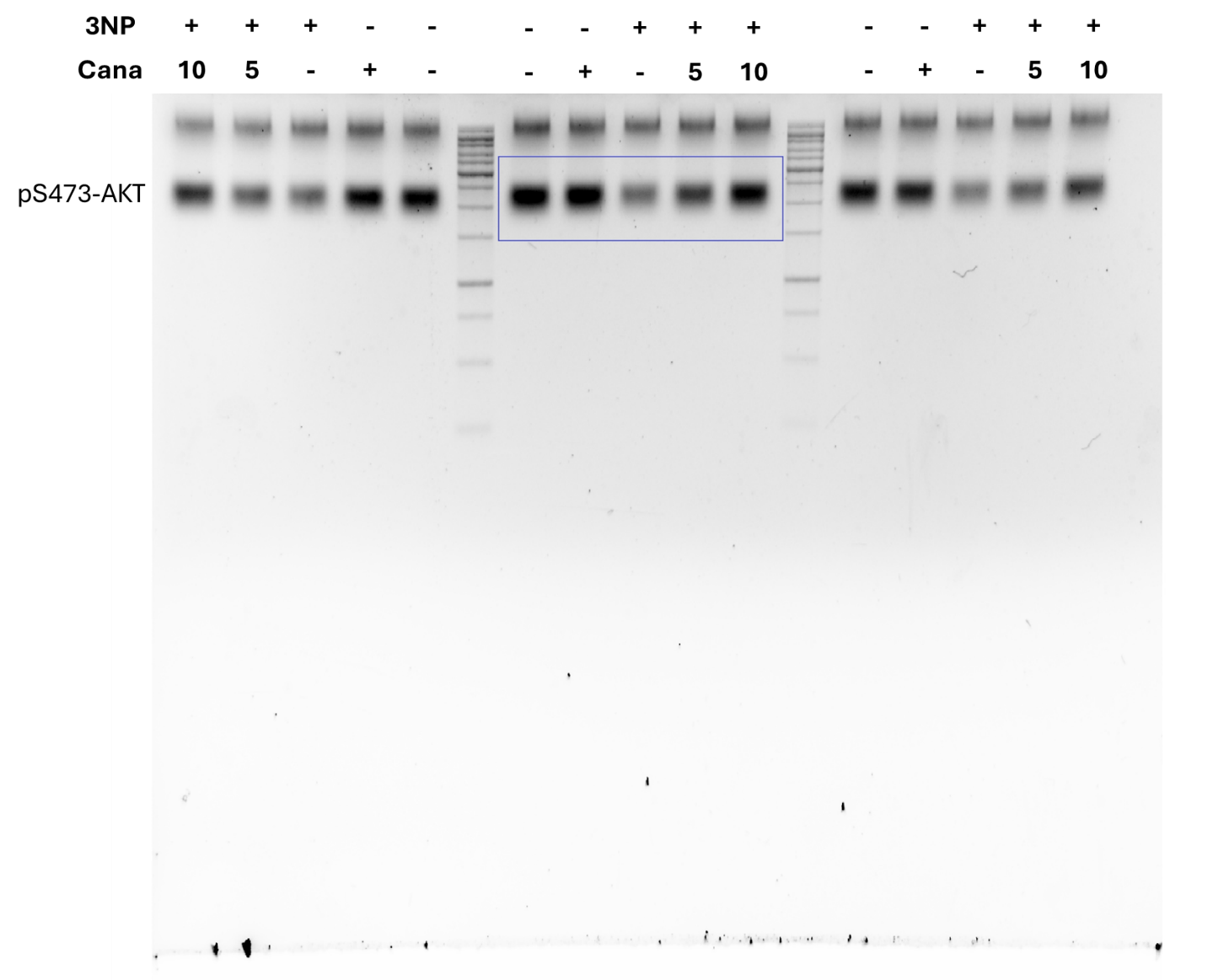


Figure 4 pS473-AKT western blot


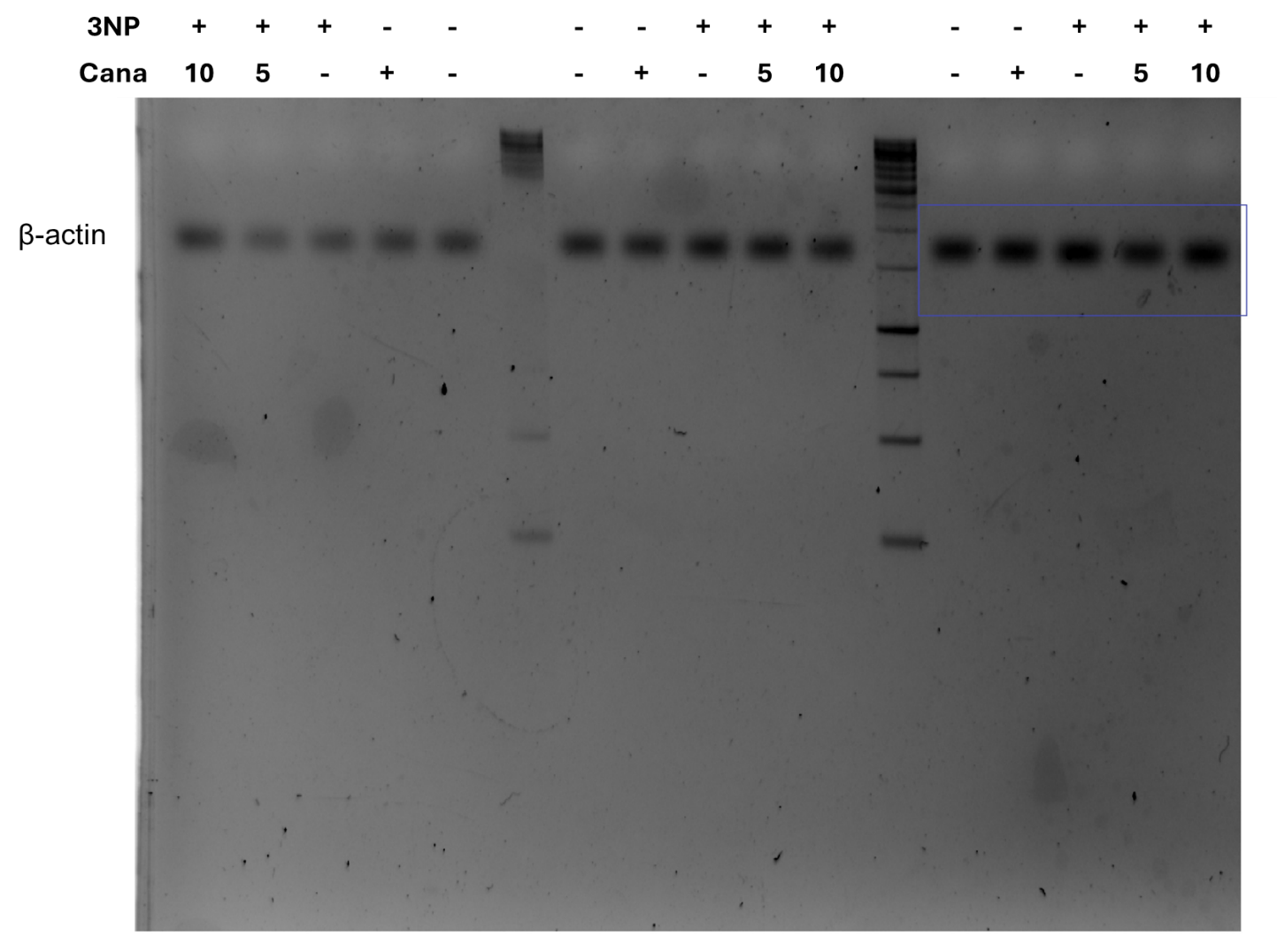

Supplement: Supplementary file 1 — (DOCX 2.66 MB) [file 210_2025_4891_MOESM1_ESM.docx]
